# 

**Published:** 1890-06

**Authors:** 


### HALL'S JOURNAL OF HEALTH

ESTABLISHED 1854

CONTENTS.

VOL. 37. No. 6.

| | PAGE. |
|---|---|
| Nature's Demands | 121 |
| The Liver | 123 |
| Botch Bosses | 124 |
| Infection in Chewing Gum | 126 |
| The Cure of Consumption | 126 |
| A cheap Filter | 127 |
| Mesmerism | 128 |
| Hygiene of Laughter | 128 |
| Recent Floods | 129 |
| Valor and Skill in the Civil War | 129 |
| The Working Girl | 130 |
| The Human Will | 131 |
| Well Water | 131 |
| Defective Sense | 132 |
| Hunger | 133 |
| A Cause of Disease | 134 |
| Sherry Wines | 135 |
| The Acids of Fruits | 135 |
| Tea Making | 136 |
| The Skin | 137 |
| Dr. Mary Walker | 137 |
| The Regeneration of the Body | 138 |
| Whitewash for Ceilings | 138 |
| Household | 139 |
| Miscellaneous | 141 |
| Literary | 143 |
| The Alpine Rose | 144 |

INDEX TO ADVERTISEMENTS OF HALF A PAGE AND OVER.

| | PAGE. |
|---|---|
| Herba-Vita Remedy Co. | I |
| Celluloid | II |
| Hollow Suppositories | III |
| Christ Before Pilate | IV |
| Hall's Journal of Health | V |
| Goldsmith's Pianos | V |
| E. C. Morris & Co. | VI |
| Dennin's Certain Cure | VII |
| Revised Premium List | VIII |
| Historical Publishing Co. | IX |
| Hospital Remedy Co. | IX |
| Herba-Vita Remedy Co. | X |

